# REFRESH-daily: a short 10-item questionnaire for evaluating behavior change towards healthier and more environmentally sustainable diets

**DOI:** 10.1186/s12966-026-01901-4

**Published:** 2026-04-01

**Authors:** Ujué Fresán, Anna Boronat, Joren Buekers, Rafael de la Torre, Laura M. König, Guillaume Chevance

**Affiliations:** 1 Institute of Agrifood Research and Technology (IRTA), Sustainability in Biosystems Research Program, Torre Marimon, Caldes de Montbui, Barcelona, 08140 Spain; 2 ISGlobal, Barcelona, Spain; 3 Integrative Pharmacology and Systems Neurosciences Research Group, Neurosciences Research Program, Hospital del Mar Research Institute (HMRI), Carrer Doctor Aiguader 88, 08003 Barcelona, Spain; 4 Department of Medicine and Life Sciences (MELIS), Universitat Pompeu Fabra (UPF), Barcelona, Spain; 5 CIBER Epidemiología y Salud Pública (CIBERESP), Madrid, Spain; 6 CIBER Fisiopatologia de La Obesidad y Nutrición (CIBEROBN), Madrid, Spain; 7 Faculty of Psychology, University of Vienna, Vienna, Austria; 8 Univ Rennes, EHESP, Inserm, Irset [(Institut de recherche en santé, environnement et travail)] - UMR_S 1085, F-35000, Rennes, France

**Keywords:** Dietary assessment, Sustainable diets, Validation study, Environmental impact, LCA, Dietary screener

## Abstract

**Background:**

Short dietary questionnaires for measuring environmentally sustainable healthy diets in daily life are scarce. REFRESH-daily (Rapid Evaluation FoR Environmentally Sustainable Healthy diets daily) is a brief 10-item questionnaire developed to fill this gap. It is administered daily and aggregates intake data to capture weekly dietary patterns and its alignment with dietary recommendations. This study aimed to evaluate its internal consistency, relative validity, construct validity, as well as end-user acceptability.

**Methods:**

A validation study was conducted among 106 adults in Spain. Participants completed REFRESH-daily for seven days via a mobile application, alongside a 7-day digital food diary as the reference method. Internal consistency was assessed using Cronbach’s alpha. Relative validity was evaluated through multi-level Bland–Altman analyses comparing REFRESH-daily with food diary data. Construct validity was tested by examining associations between REFRESH-daily score and food consumption, nutrient intake, estimated health risks based on the Global Burden of Disease framework, and environmental impact via Life Cycle Assessment methodology. Likert-scales were used to assess acceptability.

**Results:**

REFRESH-daily showed good internal consistency (Cronbach’s alpha = 0.71). Relative validity analyses showed a small mean difference of 0.3 points (out of 10) between REFRESH-daily and food diary scores. Higher REFRESH-daily scores were associated with greater intake of whole plant-based foods, health-promoting nutrients, lower estimated disease risks, and reduced environmental impacts. User acceptability was high.

**Conclusions:**

REFRESH-daily is a valid, reliable and user-friendly tool for assessing alignment with environmentally sustainable healthy diets. Its ease of use and sensitivity to subtle dietary shifts make it particularly well-suited for the integration into behavioral dietary interventions serving as a key tool for tracking progress and informing feedback in research and applied settings.

**Supplementary Information:**

The online version contains supplementary material available at 10.1186/s12966-026-01901-4.

## Introduction

Diets in high-income countries are often characterized by excessive consumption of animal-based foods and products high in sugar, salt, and unhealthy fats, alongside with inadequate intake of healthier foods such as fruits, vegetables, legumes, nuts, and whole grains [1, 2]. These prevailing dietary patterns contribute significantly to the escalating global rise in chronic non-communicable diseases and impose heavy environmental burdens, including resource depletion and high ecological impact [1–3]. Transitioning towards whole plant-based diets is increasingly recognized as a key strategy for improving both human health and environmental sustainability [2, 4, 5]. This is also reflected in the fact that several countries, including Spain, recently updated their dietary guidelines to address environmental sustainability more strongly [6].

To support this dietary transition, various behavior change interventions have been designed to promote healthier diets with lower environmental impacts [7–9]. Evaluating the effectiveness of such interventions in changing dietary behavior requires appropriate dietary assessment tools, making the selection of these tools a critical step [10]. Traditional methods, such as long food frequency questionnaire (FFQ) or food diary, provide detailed and accurate records of food consumption. However, their significant burden on participants, practitioners, and researchers makes them less practical for repeated assessments, especially when tracking behavior change over time [11]. In contrast, brief dietary questionnaires often compromise data accuracy, but are more practical for repeated assessments [12].

Several tools have been introduced to monitor dietary changes over time, such as the mini-EAT or Rapid Prime Diet Quality Score Screener. However they fail to address critical components of environmentally sustainable diets, such as meats [13] or total dairy products [14]. REFRESH (Rapid Evaluation FoR Environmentally Sustainable Healthy diets), a 10-item screener, was specifically designed to assess key food groups associated with environmentally sustainable and healthy diets. Yet, it serves primarily as a diagnostic tool to identify dietary inadequacies during a single consultation, but it was not developed to monitor precise dietary changes over time [15].

Developed as an extension of the original REFRESH tool, Fresán et al. customized it to design a daily monitoring instrument for evaluating interventions targeting environmentally sustainable healthy diets [16]. Unlike REFRESH, which frames questions around a typical week and uses a binary scoring system for its 10 items, this new instrument -named REFRESH-daily (Rapid Evaluation FoR Environmentally Sustainable Healthy diets daily)- quantifies the consumption of the same key food groups but in a daily basis and employs a graded scoring system for each of them. This refined approach allows for the detection of subtle behavioral changes over time and on specific days. The acceptability of REFRESH-daily was asserted in the previous pilot intervention for longitudinal assessments [16], but this tool has not yet undergone proper validation against a reference method.

The current study aimed to test the validity of REFRESH-daily. Specifically, the primary objective of this study was to evaluate the inter-item reliability of REFRESH-daily, its relative validity in capturing food consumption compared to food diary, and its construct validity in identifying dietary patterns aligned with both healthiness and environmental sustainability. As a secondary objective, we re-evaluated REFRESH-daily’s acceptability in a larger number of participants (compared to the initial pilot study of *N =* 12), thereby ensuring greater confidence in the tool’s user-friendliness and feasibility for large-scale applications.

## Material and methods

### Study design

The present validation study was conducted between May and July 2023. It was approved by the Comité de Ética de la Investigación con Medicamentos del Parc de Salut Mar (Observational study number 2023/10861) on Barcelona, May 10, 2023. All participants provided written informed consent prior to participating in the study.

Participants were involved in the study for nine days. On the first day, participants completed an online baseline questionnaire via the EUSurvey platform, collecting sociodemographic, lifestyle, and health-related data. Beginning on the following Monday, participants used a General Data Protection Regulation (GDPR)-compliant smartphone application (*m-Path*) to maintain a 7-day digital food diary where they were asked to indicate all the food consumed throughout each day. This dietary data served as the primary source of dietary information (i.e., reference method). Additionally, participants were prompted to complete REFRESH-daily within the same app each evening of the week. On the ninth day, participants completed a brief feedback questionnaire on the acceptability and usability of REFRESH-daily, again using the EUSurvey platform.

### Study population

Potential participants were recruited mainly via social media platforms. A post describing the study objectives, planned tasks, and inclusion criteria was published on the first author’s Instagram profile. In addition, similar information was disseminated via email through a database of individuals who had previously participated in nutrition-related studies conducted by the research group. Interested individuals received detailed study information and, upon expressing interest, were invited to complete an online screening survey. Inclusion criteria were as follows: (i) age 18 years or older; (ii) fluent in Spanish, both written and spoken; (iii) ownership of a mobile phone compatible with smartphone applications; (iv) absence of any active illness that could potentially impact eating behaviors (e.g., anorexia nervosa, bulimia nervosa, binge eating, cancer), and (v) free of cognitive or memory impairments. All criteria were self-reported by participants.

A total of 249 individuals expressed interest in participating in the study, all of whom met the inclusion criteria. From this pool, 155 individuals were invited to participate, based on sample size recommendations for dietary questionnaire validation studies—Willett and Lenhart [17] suggest a range of 100–200 participants, while Cade et al. [11] consider a minimum of 50 to be sufficient. The selection process aimed to ensure diversity in sex, age, and geographic location within Spain, thereby enhancing the study’s external validity. Ultimately, 108 individuals provided informed consent and were initially enrolled in the study. However, two participants were excluded from the analysis due to failure to complete food diary. Consequently, data was obtained from 106 participants (see Flow Chart in Additional File 1), yielding a total of 742 daily food diaries.

### REFRESH-daily

REFRESH-daily is a 10-item dietary questionnaire developed to evaluate the daily consumption of the food groups that are crucial for both dietary healthiness and environmental sustainability, particularly in high-income countries [2, 15, 16]: 1) red and processed meat; 2) white meat, fish, and eggs; 3) dairy products; 4) legumes and derivatives; 5) fruits and vegetables; 6) nuts and seeds; 7) sugary, salty, and ultraprocessed foods; 8) beverages such as sodas, juices, and energy drinks; 9) grains; and 10) added fats/oils. The current questionnaire builds upon a previous version that was used in a pilot intervention [16]. Participants who tested REFRESH-daily in the pilot trial praised its clarity, user-friendliness, and minimal time requirements, and also provided suggestions for refinement [16]. Based on those insights, the tool was refined before this validation study, mainly incorporating the option of no consumption for grains and added fats.

Participants reported their daily consumption in 0.5-serving increments, ranging from 0 to more than 5 servings, except for grains and added fats. For these two groups, participants instead indicated the proportion of servings of whole grains within total cereal intake, and virgin olive oil (representing unsaturated and non-refined added fats in the Spanish context) within total added fats. Alternatively, they could report no consumption of either. This ratio-based approach prioritized healthy food choices over strict quantity recommendations, as recommended carbohydrate and fat amounts vary according to individual energy needs. To aid in accurate reporting, the REFRESH-daily questionnaire provides examples of specific foods gathered in the assessed food groups, serving sizes in household measurements, and calculation instructions for grains and added fats. Screenshots illustrating REFRESH-daily can be found in Additional File 2, and an English translation is provided in Additional file 3.

In addition to assessing intake of each targeted food group separately, REFRESH-daily was also designed to evaluate the overall dietary adequacy by combining data from all food groups. To achieve this, we developed the REFRESH-daily scoring system, that captures the weekly environmental sustainability and healthiness of the whole diet based on the consumption of those 10 food groups. The use of a weekly dietary score, rather than a daily one, is justified by the fact that dietary intake on a given day can be balanced by intake on other days. For instance, consuming three servings of dairy products on one day may be acceptable if no dairy is consumed during the rest of the week. The REFRESH-daily score averages the daily consumption of each food group at the week level. The average daily consumption of each food group is scored on a scale from 0 to 1 point, with 1 point allocated to a consumption in line with the recommendations for an environmentally sustainable healthy diet, 0 points for consumption exceeding the limits for such a diet, and a proportional score for intermediate consumption levels. Table 1 shows the scoring criteria and the rationale, supported by up-to-date scientific evidence [2, 18–37]. The REFRESH-daily score is the sum of that of each individual item, ranging from 0 (lowest adherence to an environmentally sustainable healthy diet) to 10 (highest -best- adherence).

**Table 1:** Scoring criteria for the composite dietary score

Food groups	Scoring criteria^a^		Rationale
	**0 points**	**1 points**	
Meat, fish, and egg^b^	≥ 2 serv/d	≤ 1 serv/d	In replacement studies, substituting animal protein foods with plant-based options has been shown to reduce the risk of various chronic conditions, such as type 2 diabetes, cardiovascular disease, and even mortality [18]. Additionally, plant-based protein foods, such as legumes, have a significantly lower environmental impact compared to any animal-derived proteins [19]. Ultimately, reducing animal protein consumption and increasing plant-based protein intake minimizes the environmental impact of our diets [20, 21]. A daily serving of meat, fish, or eggs can fit within a healthy, sustainable diet, with red and processed meats limited to one serving per week [22]. Therefore, 1 point is allocated for one serving per day or less of any of these food groups, and 0 points for two or more servings per day
Red and processed meat	≥ 2 serv/w	0 serv/w	Scientific literature clearly establishes the detrimental health effects of processed meat, and to a lesser degree, red meat. Consumption of these meats shows a linear relationship with increased total mortality and other health risks in long-term consumers [2, 23, 24]. Furthermore, red meat (beef, lamb, pork) has the highest environmental impact of any protein-rich food, significantly exceeding that of other sources [19]. Therefore, for a healthy and environmentally sustainable diet, the optimal intake of red and processed meat may be 0 g/day [2]. While consuming one 100 g serving of red meat per week might be compatible with a sustainable, healthy diet, even small increases beyond this level would likely exceed environmental targets [2]. Therefore, 1 point is allocated to no consumption, and 0 points to two or more servings per week
Dairy products	≥ 2 serv/d	≤ 1 serv/d	Both the updated evaluation of the Global Burden of Disease and the Diet Impact Assessment model of the World Health Organization have reported the mixed effects of dairy products on human health [24, 27]. While dairy products have traditionally been regarded as a crucial source of calcium, this mineral can be obtained optimally from various plant-based foods [2]. Furthermore, dairy products are among the foods with the highest environmental impacts [19]. Climate change mitigation strategies advocate for reducing not only meat but also dairy product consumption [25, 26]. It is suggested that optimal dairy product intake should be at the lower end of the 0–500 ml (milk equivalents) range. Nevertheless, consuming 2 servings of dairy products daily in an omnivorous diet would exceed planetary boundaries [2], prompting recommendations to limit their consumption to a maximum of 1 serving per day [19]. Therefore, 1 point is allocated for consuming one serving or less per day of dairy products, and 0 points for consuming more 2 or more servings per day
Legume & derivatives	0 serv/d	≥ 1 serv/d	A large body of evidence highlights the health benefits of replacing animal foods with plant-based protein sources [2, 18]. The Global Burden of Disease study suggests consuming at least 100–110 g of legumes per day [27], although further benefits have been reported until 150 g per day [23]. Legumes, including sustainably grown soy products, are the protein-rich foods with the lowest environmental impact [19]. Therefore, legumes should be the main protein source in our diet for both human and planetary health. At least one serving of legumes per day is recommended for an environmentally sustainable healthy diet [2]. Thus, 1 point is allocated for consuming one or more servings of legumes per day, and 0 points for no consumption
Fruit & vegetables	0 serv/d	≥ 5 serv/d	Due to the well-stablished health benefits [2, 27] and low environmental impact of fruits and vegetables [19], at least five servings of fruits and vegetable (in total) should be consumed per day [24, 27]. Thus, 1 point is allocated to 5 or more servings per day, and 0 points to no consumption
Nuts & seeds	0 serv/d OR ≥ 4 serv/d	1–2 serv/d	Daily consumption of nuts (including peanuts), rich in proteins and healthy fats, is recommended due to their numerous health benefits. Even small amounts, such as 25–30 g, can provide significant health benefits [2, 24, 27]. While certain agricultural practices associated with some types of nuts have been noted to have relatively high environmental impacts in specific categories (for example, while almond production is water-consumptive, their carbon footprint could be very low [19]), the overall impact is moderate. Overall, their consumption, especially considering their low serving size, does not necessarily lead to an increased dietary environmental impact, particularly if consumed as a substitute for animal-based proteins [19]. Therefore, 1 point is assigned for one to two servings per day, and 0 points for no consumption or four or more servings per day
Sugary, salty and ultraprocessed foods	≥ 2 serv/w	0 serv/w	The environmental impacts of foods are determined primarily by their ingredient composition rather than their level of processing [28]. Therefore, the variability of their environmental impacts is high within this group. Conversely, the detrimental health effects of these products are well-established, and their consumption should be minimized as much as possible [29]. Therefore, due to their negative health impacts, 1 point is awarded for no consumption, and 0 points for consuming two or more servings per week
Sodas, juices and energy drinks	≥ 2 serv/w	0 serv/w	Intakes of added sugars (such as those in sodas) and free sugars (such as those in fruit juices) should be minimized as much as possible as part of a nutritionally adequate diet. Indeed, scientific evidence has not allowed for the establishment of a tolerable upper intake level for dietary added or free sugars [37]. Diet soft drinks are also not considered a healthy option [30]. Focusing on energy drinks, the Scientific Committee of the Spanish Agency for Food Safety and Nutrition recommended avoiding energy drinks due to their potential detrimental health effects [36]. Additionally, these products are usually sold in single-use plastic bottles or aluminum cans, with only a small percentage being recycled, thus exacerbating environmental degradation [31]. Therefore, 1 point is attributed to no consumption and O points for two or more servings per week
Cereals	0	≥ 80% whole grains	Although the environmental impacts of whole grains and refined options are largely similar [28, 32], both versions significantly differ in their health effects. The health benefits of whole grains are well-documented, making them a preferable choice over refined versions [2, 24, 27]. Establishing a specific quantity of whole grain consumption may not be appropriate for a dietary screener, as recommended carbohydrate intake varies based on individual energy needs [2]. Therefore, rather than focusing on the total amount of whole grains consumed, REFRESH-daily emphasizes prioritizing them over refined grains, highlighting healthy choices over quantity. Therefore, 1 point is attributed for consuming at least 80% of grains as whole grains, and 0 points for no whole grain consumption
Added fats	0	≥ 80% unsaturated unrefined oil	Unsaturated and non-refined oils should be prioritized over other added dietary fats due to their health benefits [2]. Among the available added fat options in Spain—such as virgin olive oil, refined olive oil, refined sunflower oil, margarine, butter, and lard—virgin olive oil (extra virgin or not) has been identified as the healthiest choice in our context [33]. From an environmental perspective, plant-based fats generally have lower environmental impacts compared to animal fats [19, 34]. Focusing on the plant-based oils available in Spain, olive oil has high land and water needs, while sunflower is associated with high levels of acidification and eutrophication [35]. Nevertheless, citing directly from a recent report by the International Union for Conservation of Nature (IUCN) “From an environmental point of view, talking about ‘better’ or ‘worse’ [oil] crops does not make sense, as this depends on which environmental context is highlighted and how crops are managed” [35]. Altogether, virgin (extra or not) olive oil could be regarded as the optimal option in our context due to its health benefits Similar to the approach utilized with whole grains, REFRESH proposes a ratio approach to emphasize better options over quantity, recognizing that recommended fat intake amounts vary according to individual energy needs. Therefore, 1 point is attributed to using virgin olive oil for at least 80% of total added fats, and 0 points to no virgin olive oil consumption

^a^Each item is continously scored from 0 to 1 point, according to the average consumption over the assessed week. For instance, the scoring of fruits and vegetables was: 0 points for no consumption of fruit and vegetables, and 0.25, 0.5, 0.75, and 1 points for the average consumption of 1.25, 2.5, 3.75, and 5 or more servings per day, respectively

^b^Include data of two items of the eating behavior questionnaire: i) red and processed meats, and 2) white meat, fish and eggs

#### Sensitivity analysis categorizing grains and added fats consumption

During initial testing of REFRESH-daily in the previous pilot intervention, some participants suggested providing categories of consumption of grains and added fats instead of requesting the specific percentage for simplicity [16]. To test the validity of this option, we conducted a sensitivity analysis, categorizing daily consumption of those percentual food groups into four predefined ranges: category 1 (0–24%), category 2 (25–49%), category 3 (50–74%), and category 4 (≥ 75%). The average weekly score for each food group was calculated by first averaging the daily category values across the week. Scores were then assigned on a scale from 0 to 1, with 0 points corresponding to the lowest category, 1 point to the highest, and proportional values assigned to intermediate values.

### Food diary: reference method

A 7-day food diary, implemented via the same mobile-phone application, served as the reference method to assess the validity of REFRESH-daily. This dietary assessment method required participants to record all consumed foods and beverages, estimating quantities over a specified period. This method has been widely recognized as a reliable reference for validating dietary assessment tools [38, 39]. Food diary was preferred over other common methods, such as FFQ, because the diary captures specific quantities over a short period, making them a more precise reference standard for the daily consumption targeted in REFRESH-daily. In contrast, FFQ only semi-quantitatively estimates habitual, long-term intake. A 7-day period was chosen to align with the weekly score generated by REFRESH-daily and because a full-week food diary captures both weekday and weekend variability, providing more reliable estimates of intake than shorter records such as 3-day food diary.

Participants were instructed to complete the food diary daily over seven consecutive days using an open-ended entry format accessed via a dedicated button on the app's home screen. The digital food diary interface provided clear instructions and illustrative examples to guide participants for an accurate and consistent data entry. Participants were encouraged to record their food intake immediately after consumption to minimize recall bias, although they were allowed to enter the data at any time for their convenience.

For each day, household measures reported in the diaries were converted to quantities by a nutritionist to ensure accurate quantification of dietary intake. Mixed dishes were meticulously broken down into individual ingredients based on standard household recipes. Those recipes were developed prospectively as mixed dishes (e.g., Spanish potato omelette) first appeared in the food diaries. Initial recipe drafts were generated with the assistance of artificial intelligence, and subsequently verified and finalized by a nutritionist, ensuring accurate ingredient lists and fixed proportions before use. Once defined, each recipe was applied consistently to subsequent entries of the same dish and only adjusted when participants reported specific variations (e.g., Spanish potato omelette with green peppers).

#### Food groups consumption assessment

Food quantities were converted into servings of the corresponding food groups assessed by REFRESH-daily. Standard adult serving sizes were obtained from a Spanish Clinical Nutrition Manual [40]. The proportion of whole grains relative to total grain intake and the proportion of virgin olive oil relative to total added fats were subsequently computed. Finally, the average daily consumption for each food group was calculated for the 7-day assessment period.

#### Nutrient intake assessment

Energy, macronutrient and micronutrient intakes were estimated through a detailed analysis of daily consumed foods. Supplements were not accounted for in the nutrient estimation. The Nutritics® Research Edition v6.01 software was used to estimate energy and nutrient intake. This process ensured accurate assessment of the components. Specifically, we estimated total energy intake, macronutrients (protein, total fat, monounsaturated fat, polyunsaturated fat—including omega-3 and omega-6, saturated fat, trans fat, carbohydrates, fiber, and free sugars), minerals (sodium, calcium, iron, zinc, potassium), and vitamins (A, riboflavin, folate, B_12_, C, D, and E). These nutrients were selected because: (i) they can be measured with high accuracy in a 7‑day weighed food diary; (ii) they exhibit predictable responses to changes in overall diet quality (healthier dietary patterns are typically characterized by higher intakes of dietary fiber and polyunsaturated fatty acids, while being lower in saturated and trans fats, sodium, and free or added sugars [41, 42]); and (iii) several of these nutrients are particularly critical in plant-based diets, which are generally considered more environmentally sustainable (i.e., iron, zinc, calcium, omega-3 fatty acids, and vitamins B2 (riboflavin), B12, C, and D) [43]. Average daily energy and nutrient intakes were calculated by averaging the intake across the 7-day period.

#### Dietary healthiness assessment

To evaluate the healthiness of the overall diets, we estimated the relative risk (RR) of four major diet-related diseases, including colon and rectal cancer, type 2 diabetes mellitus, ischemic heart disease, and ischemic stroke, based on average daily intake over the week. For this purpose, we utilized data from the GBD framework, which measures the association between daily consumption of specific food groups and nutrients, and disease outcomes using RR estimates. The 2021 GBD reports the RR values as the mean RR between two consumption levels, with no consumption serving as the reference (RR = 1). Assuming linear relationships between dietary components and health outcomes [44–46], we calculated the RR difference per gram of each specific dietary risk factor. For health-promoting factors, this linear relationship was assumed to hold up to the theoretical minimum risk exposure level (TMREL), beyond which no additional benefits were expected. In contrast, no threshold was applied for detrimental factors. As the GBD study provides TMREL values as a range [41], we used the average value [42]. To estimate the total dietary RR for each disease, we assumed that the effects of the dietary risk factors were multiplicative and independent, accounting for mediation [23, 46]. Notably, the effect of milk on colon and rectal cancer is fully mediated through calcium [27], and the source of fiber are other dietary risk factors (fruits, vegetables, whole grains, legumes). Therefore, milk and fiber were excluded from that specific analysis. Additional File 4 details the health outcomes assessed, the contributing dietary risk factors, corresponding RR values, and TMREL values.

#### Environmental impact assessment

The dietary environmental impact was assessed using life cycle assessment (LCA) methodology, following the ISO 14040/44:2006 norm [47, 48]. The functional unit was defined as the daily diet in mass-based units, encompassing all recorded foods. System boundaries were established from cradle to fork, including pre- and on-farm activities, processing, packaging, transportation, storage, as well as cooking. Food loss and waste within the system boundaries, as well as packaging waste management, were accounted for.

Agribalyse v3.1.1 served as the primary life cycle inventory (LCI) database. It was selected for its extensive coverage of hundreds of food items, including processed and ready-to-eat products, essential for assessing the wide variety of foods identified. Economic allocation was primarily applied in that database, with biophysical allocation for dairy husbandry and mass allocation for cheese production. See [49] for further details of this library. Each food item reported in the food diary was matched to its corresponding LCI in the Agribalyse database. Adjustments were then made to tailor the LCI data to the specific needs of this study, primarily concerning recipes, packaging materials and cooking methods. Subsequently, LCI characterization was conducted using the ReCiPe 2016 v1.1 method, employing a hierarchist perspective, both at mid- and end-point [50]. Midpoint indicators focus on single environmental problems, whereas endpoint indicators show the environmental impacts on higher aggregation levels, which are three major areas of protection, namely human health, biodiversity, and resource scarcity. In line with ISO 14040/44:2006 recommendations for transparency, all environmental categories were reported, encompassing both midpoint and endpoint indicators. This study primarily focused on the three endpoint indicators, as converting midpoints to endpoints simplifies the interpretation of the results. Additionally, the 18 midpoint environmental indicators (i.e., global warming, stratospheric ozone depletion, ionizing radiation, ozone formation-human health, fine particulate matter formation, ozone formation-terrestrial ecosystems, terrestrial acidification, freshwater eutrophication, marine eutrophication, terrestrial ecotoxicity, freshwater ecotoxicity, marine ecotoxicity, human carcinogenic toxicity, human non-carcinogenic toxicity, land use, mineral resource scarcity, fossil resource scarcity, and water consumption) on which those areas of protection are based on were individually assessed. Similar to the evaluation of food groups and nutrients, the average daily environmental impact according to each environmental indicator along the week was calculated.

###  Acceptability and usability of REFRESH-daily

An online questionnaire was developed to assess user acceptability and usability of REFRESH-daily based on principles from the unified theory of acceptance and use of technology model. The questionnaire included items rated on a 0–10 point Likert scale to assess the friendliness of the app-based questionnaire, the clarity and adequacy of information provided, the simplicity of responding, the time burden, and the overall ability of the tool to capture daily dietary intake (Additional File 5).

### Statistical analyses

#### Study population

Descriptive statistics were used to characterize the sample's socio-demographic, health-related, and lifestyle-related factors.

#### Inter-item reliability of REFRESH-daily

We assessed the inter-item reliability (i.e., internal consistency) of REFRESH-daily using Cronbach's alpha using weekly scores [51]. This coefficient, ranging from 0 to 1, measures the extent to which all items on the scale assess the same underlying concept—in our case, environmentally sustainable healthy diets. Generally, values between 0.70 and 0.95 indicate acceptable internal consistency [52]. To ensure that all items contributed positively to the scale, we conducted an item analysis. This involved sequentially removing each item and recalculating Cronbach's alpha. Items that, when removed, increased the alpha value were considered redundant and potentially detrimental to the scale's reliability. Conversely, items whose removal decreased the alpha value were deemed essential for maintaining the scale's reliability.

####  Relative validity of REFRESH-daily

We assessed the relative validity of REFRESH-daily using the food diary as reference method. We assessed the absolute agreement between both methods (i.e., REFRESH-daily and food diary) for the composite score and each of the food groups gathered using a multilevel Bland–Altman analysis that accounted for the repeated daily observations nested within individuals [11].

####  Construct validity of REFRESH-daily

To assess the construct validity of the scoring system (i.e., its ability to accurately measure dietary healthiness and environmental sustainability), we conducted univariate linear regression analyses. These analyses examined the association between the REFRESH-daily derived score and the outcomes derived from food diary, such as average daily consumption of specific food groups, energy and nutrients, RR of the health outcomes, and environmental impacts of participants' diets. Statistical significance was set at p < 0.05.

####  Acceptability and usability of REFRESH-daily

Descriptive statistics were used to characterize the acceptability and usability of REFRESH-daily.

##  Results

### Study population

Table 2 presents the sociodemographic, lifestyle, and health-related characteristics of the study participants (*n =* 106). The sample was equally balanced across age groups (50% younger than 40 years, and 50% 40 years and older; mean age: 41 years, range: 19–77), genders (52% women, 48% men), and educational levels (around one-third each with secondary education, university degrees, and postgraduate qualifications). The family incomes of three quarters of participants were at least €2,000 per month. Nearly all participants (98%) were born in Spain and were living in different Autonomous Communities during the survey period. The majority reported leading healthy lifestyles, with a high proportion being non-smokers (90%), either abstaining from or consuming alcohol only occasionally (65%), and engaging in moderate to high levels of physical activity (89%). Additionally, most participants were free of chronic diseases (88%) and food allergies or intolerances (90%).

**Table 2:** Characteristics of the study population

	Study population (*n =* 106)
**Sociodemographic characteristics**	
Age (mean (min–max))	41 (19–77) years
Gender	
Female	55 (52%)
Male	51 (48%)
Other	0 (0%)
Educational level	
No studies	0 (0%)
Primary	1 (1%)
Secondary	33 (31%)
Graduate	33 (31%)
Master	29 (27%)
PhD	10 (9%)
Family income (per month after taxes)	
Less than 500€	2 (2%)
500–999€	5 (5%)
1000–1499€	8 (8%)
1500–1999€	14 (13%)
2000–2999€	27 (25%)
30,000 (0%)3999€	25 (24%)
4000€ or more	25 (24%)
Civil status	
Single	36 (34%)
Married or stable relationship	67 (63%)
Divorced	2 (2%)
Widow	0 (0%)
Other	1 (1%)
Country of origin	
Spain	104 (98%)
Other European country	2 (2%)
Autonomous Community in Spain	
Andalucía	8 (8%)
Aragón	1 (1%)
Baleares	4 (4%)
Canarias	4 (4%)
Cantabria	2 (2%)
Castilla La Mancha	2 (2%)
Castilla León	6 (6%)
Cataluña	41 (39%)
Ceuta	1 (1%)
Euskadi	6 (6%)
Extremadura	2 (2%)
Galicia	6 (6%)
Madrid	10 (9%)
Navarra	4 (4%)
Comunidad Valenciana	9 (8%)
**Lifestyle and health**	
Smoking	
Never	69 (65%)
Given up more than 6 months ago	27 (25%)
Occasionally	8 (8%)
Daily	2 (2%)
Alcohol consumption	
Never	31 (29%)
Occasionally (less than 1 serving per week)	33 (31%)
1 or 2 drinks along the week	20 (19%)
1 serving daily or almost daily	5 (5%)
2 or more servings daily or almost daily	2 (2%)
No daily, but more than 3 servings when drinking	15 (14%)
Physical activity level	
Low	11 (10%)
Medium	55 (52%)
High	40 (38%)
Chronic disease	
None	91 (86%)
Food allergy or intolerance	
None	99 (93%)
Intake of supplements	
None	69 (65%)

Data shown as n (%)

### Inter-item reliability of REFRESH-daily

The internal consistency of the REFRESH-daily yielded a Cronbach's alpha coefficient of 0.71. This value demonstrated stability, with minimal disparity observed when individual items were removed. The largest -but still minor- variations were observed when the items red and processed meats, and legumes were removed (Cronbach´s alpha coefficient = 0.63 and 0.66, respectively), as detailed in Additional File 6. Similar findings were observed in the sensitivity analysis categorizing the proportion of whole grains and virgin olive oil consumption (Additional File 6).

### Relative validity of REFRESH-daily

**Fig. 1:**
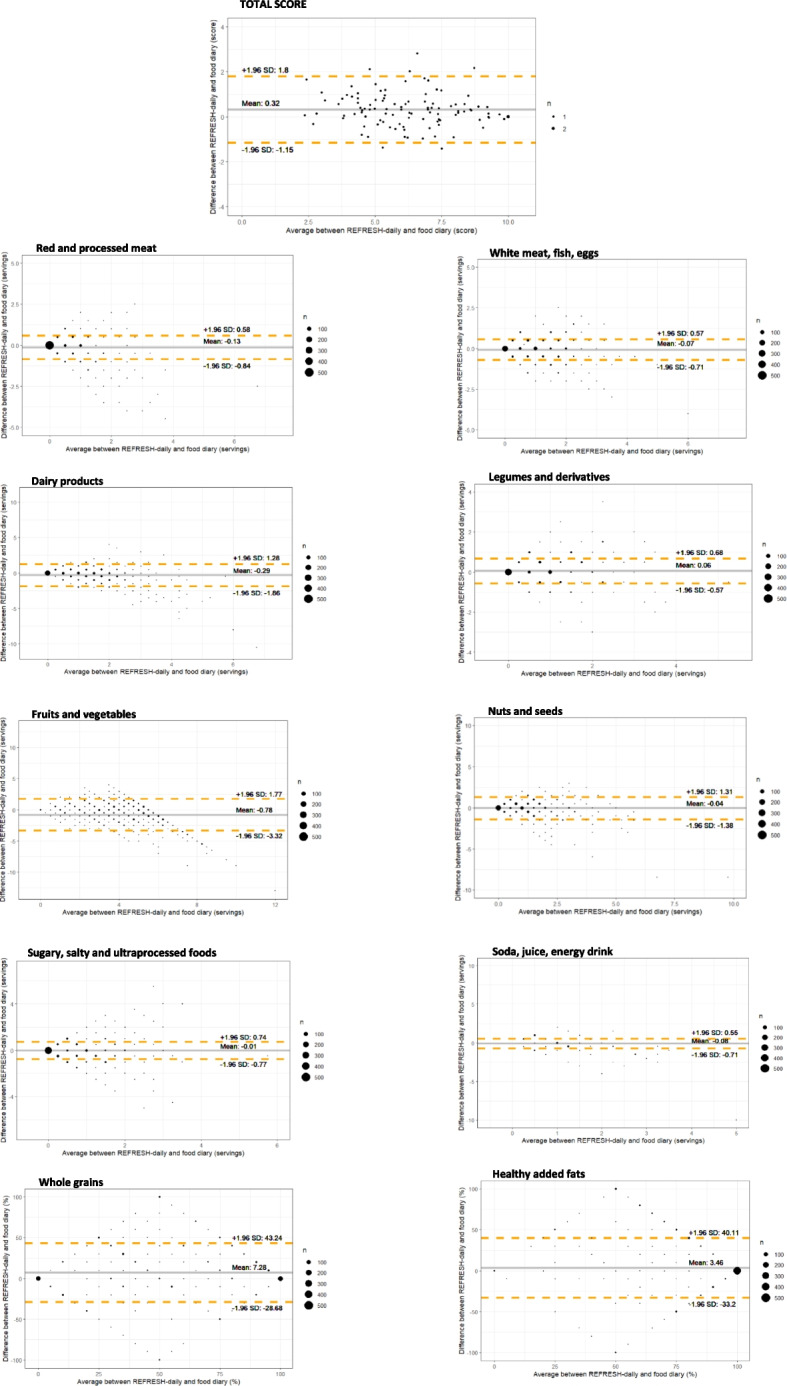
Bland–Altman plots assessing the relative validity of REFRESH-daily (total score and individual food groups), using 7-day food diary as the reference method. n: number of observations; SD: standard deviation

Bland–Altman analysis indicated that REFRESH-daily scores were, on average, 0.3 points (95% confidence interval (CI): 0.2–0.5) higher than those derived from food diary, with limits of agreement ranging from −1.2 to 1.8 points (Fig. 1). Participants scored an average of 6.4 points (range: 2.4–10) using REFRESH-daily compared to a mean of 6.1 points (range: 1.6–10) based on food diary data, on a 10-point scale.

The mean bias between methods across all targeted food groups were systematically smaller than 1 serving, ranging from −0.01 (95% CI: −0.09 to 0.06) for sugary, salty, and ultraprocessed foods to −0.8 (95% CI: −1.0 to −0.5) for fruits and vegetables. The limits of agreement were relatively narrow for all groups, with unhealthy drinks having the narrowest limits of agreement (−0.7 to 0.6 servings/day), while fruits and vegetables the widest (−3.3 to 1.8 servings/day) (Fig. 1). For food groups measured as a percentage of total consumption (whole grains and virgin olive oil), the mean differences were 7.3% (95% CI: 3.7% to 10.8%) for whole grains and 3.5% (95% CI: −0.2% to 7.1%) for virgin olive oil, though limits of agreement were broad (Fig. 1).

In the sensitivity analyses -categorizing proportion of whole grains and virgin olive oil- means scores were slightly lower for both methods compared to the main analysis (REFRESH-daily: 6.3 points (range: 2.3–10), food diary: 5.9 points (range: 1.6–10)). Nevertheless, the mean bias between methods remained consistent with the main analysis (0.3 [95% CI: 0.2–0.5] points) (Additional File 7). In relation to those food groups, most participants were classified in the same or adjacent categories when comparing REFRESH-daily responses to the 7-day food diary. Only a small proportion differed by more than one category, suggesting that REFRESH-daily captures relative intake with acceptable accuracy for these items (Additional File 7).

### Construct validity of REFRESH-daily

#### Food groups

Linear regression models showed statistically significant relationships between REFRESH-daily score and the food groups as evaluated with the reference method. Positive associations were observed with whole plant-based foods and negative associations were found between REFRESH-daily score and animal-based and unhealthy food groups (Table 3).

**Table 3:** Association between REFRESH-daily score and food groups, nutrient intake, relative risk of health outcomes, and environmental impact (linear regressions)

	**Indicators**	**Units**	**REFRESH-daily**		**Sensitivity analysis**	
			**Coeff**	***p* value**	**Coeff**	***p* value**
Food groups	Red and processed meat	Servings/day	−1.92E + 00	**< 0.001**	−1.93E + 00	**0.000**
	White meat, fish and eggs	Servings/day	−1.06E + 00	**< 0.001**	−1.06E + 00	**0.000**
	Dairy products	Servings/day	−3.90E-01	**0.013**	−3.90E-01	**0.012**
	Legumes	Servings/day	2.20E + 00	**< 0.001**	2.22E + 00	**0.000**
	Fruits and vegetables	Servings/day	5.20E-01	**< 0.001**	5.20E-01	**0.000**
	Nuts and seeds	Servings/day	7.00E-01	**< 0.001**	7.10E-01	**0.000**
	Sugary, salty and ultraprocessed foods	Servings/day	−1.97E + 00	**< 0.001**	−1.99E + 00	**< 0.001**
	Sodas, juices, energy drinks	Servings/day	−1.34E + 00	**< 0.001**	−1.35E + 00	**< 0.001**
	Whole grains	Proportion of whole grains in relation to total grains	4.00E-02	**< 0.001**	4.00E-02	**< 0.001**
	Added fats	Proportion of virgin olive oil in relation to total added fats	4.00E-02	**< 0.001**	4.00E-02	**< 0.001**
Nutrients	Total energy intake	Kcal	−1.20E-04	0.757	−1.20E-04	0.744
	Protein	g	−1.21E-02	**0.014**	−1.20E-02	**0.016**
	Total fats	g	2.89E-03	0.688	2.38E-03	0.743
	Monounsaturated fatty acids	g	1.56E-02	0.262	1.46E-02	0.296
	Polyunsaturated fatty acids	g	7.78E-02	**< 0.001**	7.76E-02	**< 0.001**
	Omega3	g	3.62E-01	**0.001**	3.66E-01	**0.001**
	Omega6	g	8.90E-02	**< 0.001**	8.93E-02	**< 0.001**
	Saturated fatty acids	g	−1.03E-01	**< 0.001**	−1.05E-01	**< 0.001**
	Trans fatty acids	g	−6.62E-01	**0.002**	−6.83E-01	**0.002**
	Carbohydrates	g	4.61E-03	0.133	4.68E-03	0.129
	Fibre	g	8.51E-02	**< 0.001**	8.56E-02	**< 0.001**
	Free sugars	g	−6.69E-02	**< 0.001**	−6.84E-02	**< 0.001**
	Sodium	mg	−1.06E-03	**< 0.001**	−1.08E-03	**< 0.001**
	Calcium	mg	4.80E-04	0.345	4.70E-04	0.361
	Iron	mg	1.80E-01	**< 0.001**	1.81E-01	**< 0.001**
	Zinc	mg	1.18E-01	0.083	1.17E-01	0.089
	Potassium	mg	6.80E-04	**< 0.001**	6.80E-04	**< 0.001**
	Vitamin A	Retinol equivalents	9.50E-04	**0.004**	9.40E-04	**< 0.001**
	Riboflavin	mg	−1.09E-01	0.754	−1.20E-01	0.732
	Folate	μg	4.60E-03	**< 0.001**	4.58E-03	**< 0.001**
	Vitamin B12	μg	−2.85E-01	**< 0.001**	−2.90E-01	**< 0.001**
	Vitamin C	mg	1.11E-02	**< 0.001**	1.11E-02	**< 0.001**
	Vitamin D	μg	−2.53E-02	0.786	−2.82E-02	0.764
	Vitamin E	mg	1.22E-01	**0.001**	1.22E-01	**0.001**
Health effect	Colom and rectal cancer relative risk	Relative risk	−4.09E + 00	**< 0.001**	−4.05E + 00	**< 0.001**
	Diabetes mellitus relative risk	Relative risk	−5.33E + 00	**< 0.001**	−5.32E + 00	**< 0.001**
	Ischemic heart disease	Relative risk	−9.22E + 00	**< 0.001**	−9.17E + 00	**< 0.001**
	Ischemic stroke	Relative risk	−9.06E + 00	**< 0.001**	−8.96E + 00	**< 0.001**
Environmental impact indicators	Human health	DALY	−1.76E + 05	**< 0.001**	−1.75E + 05	**< 0.001**
	Ecosystems	Species.yr	−3.23E + 07	**< 0.001**	−3.21E + 07	**< 0.001**
	Resources	USD2013	−6.41E + 00	**< 0.001**	−6.41E + 00	**< 0.001**
	Global warming	kg CO_2_ eq	−5.90E-01	**< 0.001**	−5.90E-01	**< 0.001**
	Stratospheric ozone depletion	kg CFC11 eq	−8.94E + 04	**< 0.001**	−8.94E + 04	**< 0.001**
	Ionizing radiation	kBq Co-60 eq	−3.61E + 00	**< 0.001**	−3.67E + 00	**< 0.001**
	Ozone formation, Human health	kg NOx eq	−7.77E + 01	**0.001**	−7.70E + 01	**0.001**
	Fine particulate matter formation	kg PM_2.5_ eq	−2.51E + 02	**< 0.001**	−2.50E + 02	**< 0.001**
	Ozone formation, Terrestrial ecosystems	kg NOx eq	−7.71E + 01	**0.001**	−7.65E + 01	**0.001**
	Terrestrial acidification	kg SO2 eq	−6.66E + 01	**< 0.001**	−6.66E + 01	**< 0.001**
	Freshwater eutrophication	kg P eq	−7.30E + 02	**0.042**	−7.29E + 02	**0.044**
	Marine eutrophication	kg N eq	−6.88E + 01	0.076	−6.64E + 01	0.090
	Terrestrial ecotoxicity	kg 1,4-DCB	1.00E-02	0.722	1.00E-02	0.672
	Freshwater ecotoxicity	kg 1,4-DCB	−2.57E + 00	0.538	−2.59E + 00	0.538
	Marine ecotoxicity	kg 1,4-DCB	−3.42E + 00	0.295	−3.44E + 00	0.295
	Human carcinogenic toxicity	kg 1,4-DCB	−1.06E + 01	**0.001**	−1.08E + 01	**0.001**
	Human non-carcinogenic toxicity	kg 1,4-DCB	−3.00E-02	0.555	−3.00E-02	0.601
	Land use	m^2^a crop eq	−4.10E-01	**0.001**	−4.10E-01	**0.001**
	Mineral resource scarcity	kg Cu eq	−2.44E + 01	0.429	−2.44E + 01	0.431
	Fossil resource scarcity	kg oil eq	−2.55E + 00	**< 0.001**	−2.55E + 00	**< 0.001**
	Blue water consumption	m^3^	2.64E + 00	**0.028**	2.76E + 00	**0.022**

Sensitivity analysis implied categorizing whole grains and added fats items in four categories (1: 0–24%; 2: 25–49%; 3: 50–74%; 4: ≥ 75%)

*p*-values < 0.05 are considered statistically significant, and are highlighted in bold

*Kcal* Kilocalories, *DALY* Disability-adjusted life years; species.yr: potential species loss per year, *USD2013* U.S. dollars (2013 value), *kg CO₂ equivalents* kilograms of carbon dioxide equivalents, *kg CFC11 equivalents* kilograms of trichlorofluoromethane equivalents, *kBq Co-60 equivalents* kilobecquerels of cobalt-60 equivalents, *kg NOx equivalents* kilograms of nitrogen oxides equivalents, *kg PM₂.₅ equivalents* kilograms of fine particulate matter (PM_2.5_) equivalents, *kg SO₂ equivalents* kilograms of sulfur dioxide equivalents, *kg P equivalents* kilograms of phosphorus equivalents, *kg N equivalents* kilograms of nitrogen equivalents, *kg 1,4-DCB* kilograms of 1,4-dichlorobenzene equivalents, *m*^2^a crop equivalents square meter-years of crop-equivalent land use, *kg Cu equivalents* kilograms of copper equivalents, *kg oil equivalents* kilograms of oil equivalents, *m*^3^ cubic meters

#### Nutrients

The REFRESH-daily score was positively and significantly associated with the intake of beneficial nutrients, including health-promoting macronutrients like polyunsaturated fatty acids (total, and both omega-3 and omega-6) and dietary fiber, as well as micronutrients such as iron, potassium, and vitamins A, B_9_ (folate), C, and E. On the other hand, the REFRESH-daily score was negatively associated with the intake of less healthy nutrients, specifically saturated and trans fatty acids, free sugars, and sodium. No significant associations were found between the REFRESH-daily score and total energy intake, total fats, monounsaturated fatty acids, carbohydrates, calcium, zinc, and vitamins B_2_ and D. The REFRESH-daily score was negatively and significantly associated with intakes of protein and vitamin B_12_ (Table 3).

#### Health outcomes

The REFRESH-daily score was significantly and negatively associated with the estimated relative risk of all four health outcomes studied, namely colon and rectal cancer, type 2 diabetes, ischemic heart disease, and ischemic stroke (Table 3).

#### Environmental impacts

Regarding environmental impacts (Table 3), significant negative associations were observed between REFRESH-daily score and the three endpoint indicators (human health, ecosystem quality, and resource availability). At the midpoint level (i.e., for each individual environmental indicator), 11 of the 18 assessed indicators showed significant negative associations, meaning higher REFRESH-daily score was linked to lower environmental burden. Six midpoint indicators showed no significant association with REFRESH-daily score (i.e., marine eutrophication, terrestrial ecotoxicity, freshwater ecotoxicity, marine ecotoxicity, non-carcinogenic human toxicity, and mineral resource scarcity). Only blue water consumption showed a significant and positive association, indicating that higher REFRESH-daily scores were associated with increased blue water consumption.

#### Sensitivity analyses

Similar findings for the four outcomes (food groups, nutrients, health outcomes and environmental impacts) were observed in the sensitivity analysis examining the proportion of whole grain and virgin olive oil consumption across categories (Table 3).

### Acceptability and usability of REFRESH-daily

The results indicate high overall satisfaction with REFRESH-daily. Participants rated the app-based questionnaire easy to use (median* =* 10, interquartile range (p25-p75) = 8–10), judged the evaluation straightforward (median* =* 10, p25-p75 = 8–10) and quick to complete (median* =* 10, p25-p75 = 9–10). Additionally, participants agreed that REFRESH-daily provided sufficient information for an accurate food classification (median* =* 8, p25-p75 = 7–10) and offered a clear understanding of their dietary intake (median* =* 8, p25-p75 = 7–9).

##  Discussion

REFRESH-daily is a concise, low-burden questionnaire designed for monitoring food intake aligned with environmentally sustainable healthy diets. Overall, the questionnaire showed good internal consistency and agreement with the reference method (i.e., the 7-day food diary). Construct validity was supported by significant and positive associations between REFRESH-daily score and greater intake of whole, plant-based foods and health-promoting nutrients, and significant and negative associations with the intake of harmful dietary components. A higher score was also associated with reduced relative risk of adverse health outcomes and lower environmental impacts. Furthermore, the tool showed good acceptability among participants.

###  Inter-item reliability

Despite addressing two distinct dietary dimensions—human health and environmental sustainability—REFRESH-daily shows satisfactory internal consistency, with coefficients similar to those of previously validated dietary assessment instruments [15, 53]. The absence of improved internal consistency upon removal of any item supports the inclusion of all components and reinforces the structural coherence and comprehensiveness of the instrument. Notably, decreases in internal consistency when removing red and processed meat and legume items highlights the central role of these protein food groups in environmentally sustainable healthy diets.

###  Relative validity

Our findings support the accuracy of REFRESH-daily in capturing actual food consumption when compared with the reference 7-day food diary. On a 10-point scale, REFRESH-daily score was only marginally higher—by 0.3 points—than that derived from the food diary, with narrow limits of agreement. This difference is notably smaller than that reported in validation studies of other brief dietary tools [54, 55]. The relative validity of individual food groups is also particularly valuable for investigating within-person daily variations [56, 57], and co-variations [58] or spillovers [59] among food groups. The unique daily administration of REFRESH-daily may minimize recall and social desirability biases typically associated to methods employing longer recall periods (e.g., during last week, lost month, last trimester), thus enhancing its accuracy in collecting dietary consumption [60]. Additionally, REFRESH-daily is based on repeated measures and thus might improve memory and reduce bias over time. On the contrary, a challenge of REFRESH-daily is that it must be completed daily—or at least on two weekdays and one weekend day [61]—to obtain a precise estimate of the composite REFRESH-daily score. Nevertheless, the tool is considered quick to complete and user-friendly. Emphasizing these strengths may help improve adherence and support the establishment of a consistent response habit during the trial period.

Focusing on individual food groups, the mean difference between REFRESH-daily and the food diary was smaller than 0.5 servings for most groups. This is particularly reassuring given that REFRESH-daily uses 0.5-serving increments. Such small differences may indicate that participants tended to round down intermediate quantities when completing REFRESH-daily, or alternatively, that food diary may slightly overestimate intake due to challenges in portion size estimation. The most notable discrepancy—though still modest—was observed for fruits and vegetables, which were underreported in REFRESH-daily by approximately 0.8 servings per day. This was accompanied by slightly wider limits of agreement. Although healthy food groups are often overreported in self-reported dietary assessments due to social desirability bias [61], similar underestimations of fruit and vegetable intake have been reported in other dietary screeners’ validation studies [62]. A plausible explanation is that small quantities of fruits and vegetables embedded in mixed dishes (e.g., sauces or toppings) may have been overlooked by participants when completing REFRESH-daily but were captured through the more detailed food diary. The wider limits of agreement may also reflect this systematic underreporting, compounded by the inherently higher frequency and volume of fruit and vegetable consumption, which contribute to greater variability in intake.

While the mean differences between methods for assessing whole grain and virgin olive oil intake were small, the limits of agreement were notably wide. These food groups are expressed as a percentage of total grains and added fat intake, respectively, including a variety of foods. This could make accurate estimation more challenging. Participants may struggle to identify all items in the group, to distinguish whole grains from refined cereals [63] or to report specific added fats used when meals were prepared by others. The composite nature of these groups, combined with varying portion sizes (e.g., bread vs. pasta), may further complicate percentage-based estimations. Nonetheless, discrepancies may also stem from limitations in food diary data, including incomplete or imprecise entries. Either way, when using REFRESH-daily in controlled studies, we recommend participant training in quantifying these specific food groups. Alternatively, employing the categories used in the sensitivity version could be considered. This approach would simplify the estimation process and potentially reduce reporting bias. However, it introduces a level of discretization absent in the original percentage estimations, which may affect the sensitivity of the assessment. Regardless, the validity of both versions of REFRESH-daily was tested and confirmed in this study.

###  Construct validity

Our validation analyses support a positive association between REFRESH-daily score and indicators of human health. First, higher REFRESH-daily score was associated with whole plant-based diets, which are widely recognized for their health benefits [64–67]. Second, the score was positively correlated with health-promoting nutrients and negatively with detrimental ones. Although the association with protein and vitamin B_12_ was negative, this did not indicate insufficient intake for higher REFRESH-daily score. Mean daily intakes of protein and vitamin B12 among the study sample exceeded the recommended requirements for the Spanish population [24]. Rather, this likely reflects the excessive consumption of protein—particularly from animal sources—among individuals scoring low on REFRESH-daily, a pattern that has been previously documented in the Spanish population [68, 69]. No associations were observed for calcium and vitamin D, while positive associations were found with iron and omega-3 fatty acids—challenging the common assumption that self-selected plant-based diets are inherently deficient in these nutrients [70]. Third, REFRESH-daily scores were associated with a lower estimated relative risk of adverse health outcomes. Although our cross-sectional data did not allow for direct assessment of health outcomes, we relied on established modeling approaches used by organizations such as the GBD study [42] and the World Health Organization [24] to estimate the health impact of dietary patterns. Longitudinal studies are needed to confirm these associations using primary health outcomes. Fourth, our findings indicate that higher REFRESH-daily score also corresponds with reduced human health damage linked to environmental pollution. This dimension is often overlooked in nutritional research, yet given the worsening environmental crisis, its inclusion is increasingly critical [69, 71].

Our findings demonstrate that REFRESH-daily is also effective in identifying environmentally sustainable dietary patterns. This validation is essential, as the assumption that healthy diets inherently result in low environmental impact is not always accurate. The environmental footprint of a healthy diet can vary—being either higher or lower than that of the average diet—depending on the specific food choices involved [21]. Similarly, diets with low environmental impact do not necessarily confer health benefits [72]. Moreover, trade-offs between environmental impact indicators have been described in the literature. For example, certain fish species may have a low carbon footprint but are sourced from overexploited stocks, raising concerns about biodiversity loss [73]. To address these complexities, we moved beyond the conventional focus on greenhouse gas emissions [74], and assessed a broad range of environmental indicators, including 3 endpoint and 18 midpoint measures. A negative significant association (higher REFRESH-daily score, lower environmental burden) was observed for 11 of the midpoint indicators. Blue water consumption was the only environmental category showing a positive association with higher score, likely due to the irrigation-dependent cultivation of some food groups positively associated with REFRESH-daily (e.g., fruits, vegetables, nuts). It is important to note that this indicator only considers blue water (ground and surface water), not green water (rain water), for which animal-based foods are major consumers [75]. While green water is not frequently characterized in LCAs, its consideration is increasingly important due to the surpassing of its planetary boundary [76]. Critically, considering both green and blue water, animal products are among the most water-demanding foods [75]. Therefore, the inclusion of green water in our analysis may have yielded different results. Regardless, the overall reduction on environmental impacts was further supported by the inverse association between REFRESH-daily score and the three endpoint indicators, reflecting lower impacts on human health, biodiversity, and resource scarcity with higher REFRESH-daily score.

###  Strengths, limitations and applicability

REFRESH-daily has been validated in a diverse cohort in terms of age, sex, and educational level, encompassing participants from different regions of Spain with potentially diverse culinary habits. While the broad scope of this sample enhances external validity, it must be acknowledged that it may not be entirely representative of the general Spanish population. Particularly notable is the high proportion of participants from Cataluña [77], likely due to recruitment channels such as the authors’ social media and internal databases, which are predominantly based in this region. Additionally, there was limited representation of individuals with a low socioeconomic status [78] and low educational level [79]. This imbalance is relevant because socioeconomic and educational status can be correlated with dietary quality and, potentially, the capacity to effectively use or engage with a detailed tool like REFRESH-daily [80, 81]. Furthermore, participants reported lower prevalence of chronic conditions [82] and smoking [83] and higher physical activity level [84] than typically observed in national surveys, suggesting a generally healthier and more health-conscious cohort, which may influence the ability to engage with REFRESH-daily.

The capacity of REFRESH-daily to capture adherence to healthy diets with low environmental impact was tested using a multifaceted approach that extended beyond traditional measures such as nutritional quality and greenhouse gas emissions. We complemented the nutritional analysis with a dietary healthiness assessment, although confirmation of this theoretically-driven health benefits on longitudinal cohort data is pending. Additionally, our environmental impact assessment incorporated 21 environmental indicators across multiple categories, allowing for a more broad and detailed analysis, accounting for potential trade-offs. Importantly, we also validated two versions of REFRESH-daily—one using percentage-based variables and the other using categorical variables for grain and added fat consumption—allowing practitioners to choose the level of detail that best fits their specific research or intervention.

Designed to align with the Spanish context, REFRESH-daily reflects local food availability and traditional dietary practices in that country. Still, the questionnaire can be easily adapted to other countries' specific needs. For instance, while virgin olive oil is central to Spanish and Italian diets, the questionnaire can be modified to highlight other unsaturated, unrefined fats, like canola oil in Nordic countries [85, 86]. Adapting the examples and clarifications within the questionnaire to reflect specificities of national gastronomy seemed essential to ensure accurate comprehension and relevance across diverse cultural contexts. Although cultural adaptation could follow a similar validation process, significant differences in the validity of REFRESH-daily are not anticipated if only minor modifications were implemented.

The utility of REFRESH-daily extends beyond behavioral interventions. Given its ability to capture both health and environmental aspects of diet in a brief and user-friendly format, REFRESH-daily may also serve as a valuable tool for national dietary surveillance, and even contribute to global monitoring efforts such as the Food System Countdown Initiative [87], which aims to track progress across multiple domains of food system transformation, including dietary health and sustainability.

##  Conclusion

REFRESH-daily has demonstrated its validity as a practical, reliable and user-friendly tool for measuring eating behaviors towards healthier and more environmentally sustainable dietary patterns. Its strong internal consistency, robust agreement with reference dietary data, and significant associations with key food groups consumption, nutrient intake, health outcomes, and environmental impacts, as well as high acceptability support its use in dietary interventions and public health research.

## Supplementary Information

Supplementary Material 1

## Data Availability

The datasets generated and analyzed during the current study are available in Open Science Framework: https://osf.io/st2an/.

## Abbreviations

REFRESH: Rapid Evaluation FoR Environmentally Sustainable Healthy diets
REFRESH-daily: Rapid Evaluation FoR Environmentally Sustainable Healthy diets daily
RR: Relative Risk
GBD: Global Burden of Disease
TMREL: Theoretical Minimum Risk Exposure Level
LCA: Life Cycle Assessment
LCI: Life Cycle Inventory
CI: Confidence Interval
p25-p75: Interquartile range
